# A self-administered, artificial intelligence (AI) platform for cognitive assessment in multiple sclerosis (MS)

**DOI:** 10.1186/s12883-020-01736-x

**Published:** 2020-05-18

**Authors:** Seyed-Mahdi Khaligh-Razavi, Maryam Sadeghi, Mahdiyeh Khanbagi, Chris Kalafatis, Seyed Massood Nabavi

**Affiliations:** 1 Cognetivity Ltd, London, UK; 2 Department of Brain and Cognitive Sciences, Cell Science Research Center, Royan Institute for Stem Cell Biology and Technology, ACECR, Tehran, Iran; 3 University of Tehran, Tehran, Iran; 4 South London & Maudsley NHS Foundation Trust, London, UK; 5 Department of Old Age Psychiatry, King’s College London, London, UK

**Keywords:** Multiple sclerosis, BICAMS, Digital biomarkers, Integrated cognitive assessment (ICA), Language-independent, Artificial intelligence (AI)

## Abstract

**Background:**

Cognitive impairment is common in patients with multiple sclerosis (MS). Accurate and repeatable measures of cognition have the potential to be used as markers of disease activity.

**Methods:**

We developed a 5-min computerized test to measure cognitive dysfunction in patients with MS. The proposed test – named the Integrated Cognitive Assessment (ICA) – is self-administered and language-independent. Ninety-one MS patients and 83 healthy controls (HC) took part in Substudy 1, in which each participant took the ICA test and the Brief International Cognitive Assessment for MS (BICAMS). We assessed ICA’s test-retest reliability, its correlation with BICAMS, its sensitivity to discriminate patients with MS from the HC group, and its accuracy in detecting cognitive dysfunction. In Substudy 2, we recruited 48 MS patients, 38 of which had received an 8-week physical and cognitive rehabilitation programme and 10 MS patients who did not. We examined the association between the level of serum neurofilament light (NfL) in these patients and their ICA scores and Symbol Digit Modalities Test (SDMT) scores pre- and post-rehabilitation.

**Results:**

The ICA demonstrated excellent test-retest reliability (*r* = 0.94), with no learning bias, and showed a high level of convergent validity with BICAMS. The ICA was sensitive in discriminating the MS patients from the HC group, and demonstrated high accuracy (AUC = 95%) in discriminating cognitively normal from cognitively impaired participants. Additionally, we found a strong association (*r* = − 0.79) between ICA score and the level of NfL in MS patients before and after rehabilitation.

**Conclusions:**

The ICA has the potential to be used as a digital marker of cognitive impairment and to monitor response to therapeutic interventions. In comparison to standard cognitive tools for MS, the ICA is shorter in duration, does not show a learning bias, and is independent of language.

## Background

Multiple sclerosis (MS) is characterized by widespread demyelination and neurodegeneration in the central nervous system [1]. Therefore, cognitive dysfunction is common in MS patients (40–70% of these patients are reported to have cognitive impairment [2]), and is associated with a higher risk of disease progression in the subsequent years [2]. Cognitive impairment can have significant negative impacts on several domains of daily living activities, such as social functioning, employment [3] and driving [4]. Despite the prevalence of cognitive impairment and its negative impact on patients’ lives, cognitive assessment is not routinely carried out for MS patients [5].

Early detection of cognitive impairment in MS could be helpful in the identification of patients at high risk of disability progression and poor clinical outcome [6]. Furthermore, cognition has the potential to be used as a marker of disease progression or treatment efficacy in MS [7, 8]. When patients report a cognitive problem, they are describing a change in function from a previous level; however, the majority of cognitive tests, due to a learning bias [9, 10], cannot be used for frequent monitoring of cognitive performance. On the other hand, neuroimaging and fluid biomarkers of disease activity [11–13], while more accurate, are less suitable for frequent monitoring of disease progression and more difficult to integrate into routine clinical practice. Here, we propose an AI-assisted digital biomarker of cognitive function, appropriate for monitoring disease activity.

There is evidence that the afferent visual system is highly vulnerable to MS [14]. Furthermore, deficit in information processing speed (IPS) is the most prevalent cognitive impairment in MS, and can affect the speed of sensory, motor and cognitive processes [15]. We designed an iPad-based rapid visual categorization task [16–18] the Integrated Cognitive Assessment (ICA), that primarily assesses IPS in visuomotor domains. The task is designed to give a sensitive, repeatable measure of IPS, and is additionally shown to be correlated with other cognitive domains, such as verbal memory and visuospatial abilities [19]. The test is software-based, self-administered and is shown to have little dependency on participants’ language, and is not confounded by participants’ varying levels of education [19].

To measure the efficacy of the proposed ICA test in detecting cognitive impairment in MS patients, we compared the ICA with the Brief International Cognitive Assessment for MS (BICAMS) [20, 21]. BICAMS is a pen-and-paper based cognitive assessment battery for detecting cognitive dysfunction in MS patients. The BICAMS battery includes tests of mental processing speed as well as visuospatial and verbal learning, and takes about 15 to 20 min to administer and score.

To further assess the validity of the ICA test as a potential digital biomarker of MS disease activity, we compared ICA test results with participants’ level of serum neurofilament light chain (NfL). NfL has been shown to be a valuable fluid biomarker of MS disease activity and treatment response [22], and is associated with clinical and MRI-related measures of disease activity and neuroaxonal damage [23]. Changes in NfL are shown to be associated with changes in global cognition and attention [24]. Furthermore, elevated baseline plasma NfL is a prognostic marker of cognitive decline and neuroimaging measures of neurodegeneration, and has similar effect sizes to baseline cerebrospinal fluid (CSF) NfL [24].

We report results for convergent validity between BICAMS and ICA, test-retest reliability, correlation between ICA score and serum NfL, the effects of repeated exposure to the tests (i.e. learning bias), sensitivity to detecting cognitive impairment, and the accuracy of the ICA in discriminating MS patients from healthy controls (HC).

## Methods

### ICA test description and the scientific rationale behind the test

**Fig. 1:**
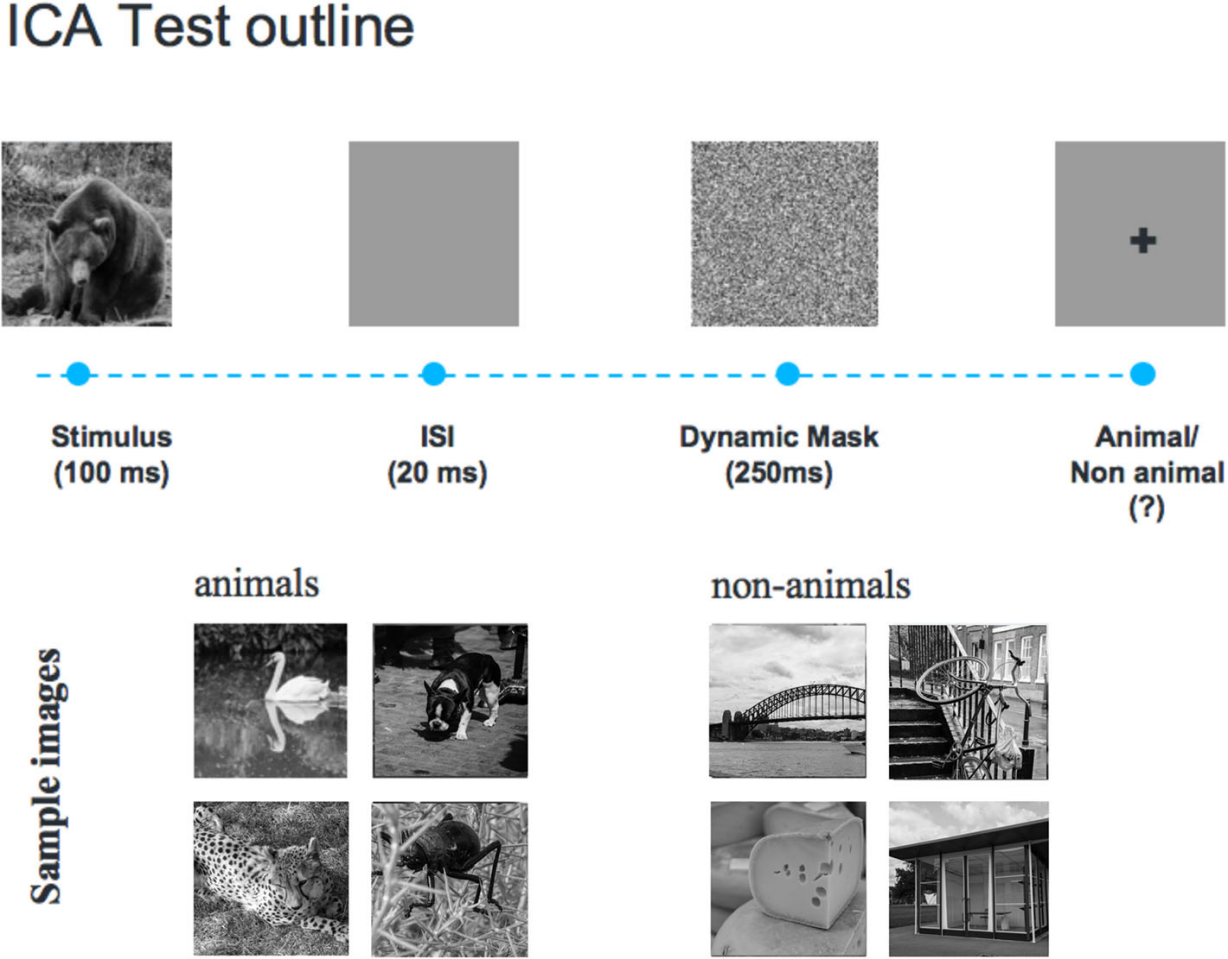
The ICA test pipeline. One hundred natural images (50 animal and 50 non-animal) with various levels of difficulty are presented to the participants. Each image is presented for 100 ms followed by 20 ms inter-stimulus interval (ISI), followed by a dynamic noisy mask (for 250 ms), followed by categorization into animal vs. non-animal. A few sample images are shown for demonstration purposes

The ICA test is a rapid visual categorization task with backward masking [17, 18, 25]. The test takes advantage of the human brain’s strong reaction to animal stimuli [25, 26]. One hundred natural images (50 animal and 50 non-animal) are carefully selected, with varying levels of difficulty, and are presented to the participants in rapid succession. Images are presented in the center of the screen at a 7° visual angle. In some images the head or body of the animal is clearly visible to the participants, which makes it easier to detect. In other images the animals are further away or otherwise presented in cluttered environments, making them more difficult to detect. A few sample images are shown in Fig. 1. We used grayscale images to remove the possibility of color blindness affecting participants’ results. Furthermore, color images can facilitate animal detection solely based on color [27, 28], without full processing of stimulus shape. This could have made the task easier and less suitable for detecting less severe cognitive dysfunctions.

The strongest categorical division represented in the human higher level visual cortex appears to be that between animates and inanimates [29, 30]. Studies also show that on average it takes about 100 ms to 120 ms for the human brain to differentiate animate from inanimate stimuli [26, 31, 32]. Following this rationale, each image is presented for 100 ms followed by a 20 millisecond inter-stimulus interval (ISI), followed by a dynamic noisy mask (for 250 ms), followed by the subject’s categorization into animal vs. non-animal (Fig. 1). Shorter ISI durations can make the animal detection task more difficult and longer durations reduce the potential use for testing purposes as it may not allow for the detection of less severe cognitive impairments. The dynamic mask is used to remove (or at least reduce) the effect of recurrent processes in the brain [33, 34]. This makes the task more challenging by reducing the ongoing recurrent neural activity that could artificially boost a subject’s performance and further reduces the chances of learning the stimuli. For more information about rapid visual categorization tasks refer to Mirzaei et al., (2013) [17].

The ICA test starts with a different set of 10 test images (5 animal, 5 non-animal) to familiarize participants with the task. These images are later removed from further analysis. If participants perform above chance (> 50%) on these 10 images, they will continue to the main task. If they perform at chance level (or below), the test instructions will be presented again, and a new set of 10 introductory images will follow. If they perform above chance in this second attempt, they will progress to the main task. If they perform below chance for the second time the test is aborted.

#### Backward masking

To construct the dynamic mask a white noise image was filtered at four different spatial scales, and the resulting images were thresholded to generate high contrast binary patterns following the procedure in Bacon-Macé and colleagues (2005) [16, 17]. For each spatial scale, four new images were generated by rotating and mirroring the original image, creating a pool of 16 images. The noisy mask used in the ICA test was a sequence of 8 images, chosen randomly from the pool, with each of the spatial scales appearing twice.

### Brief international cognitive assessment for MS (BICAMS)

The BICAMS battery consists of three standard pen-and-paper tests, measuring speed of information processing, visuospatial learning and verbal learning.

#### Symbol digit modalities test (SDMT)

The SDMT is designed to assess speed of information processing, and takes about 5 min to administer [35]. The test is formed of a simple substitution task. Using a reference key, the examinee has 90 s to pair specific numbers with given geometric figures.

#### California verbal learning test - 2nd edition (CVLT-II)

The CVLT-II test [36, 37] measures episodic verbal learning. The test begins with the examiner reading a list of the 16 words. Participants listen to the list and then report as many of the items as they can recall. Five learning trials of the CVLT-II are used in BICAMS [20], which takes about 10 min to administer.

#### Brief visual memory test – revised (BVMT-R)

The BVMT-R test assesses visuospatial learning (i.e. immediate recall) and memory (delayed recall) [38, 39]. Only learning trials of BVMT-R are included within BICAMS. Here, in three consecutive trials, six abstract shapes are presented to the participant for 10 s. After each trial, the display is removed from view and patients are asked to draw the stimuli via pencil on paper manual responses. The test takes about 5 min to administer.

### Participants

In total, 174 participants took part in Substudy 1 (Table 1): 91 patients diagnosed with multiple sclerosis (MS), and 83 healthy controls matched for age, gender and education. Fourty-eight MS patients took part in Substudy 2 (Table 2). Of all participants, 25 attended both substudies. Participants’ age varied between 18 and 65. The study was conducted according to the Declaration of Helsinki and approved by the local ethics committee at Royan Institute. Informed written consent was obtained from all participants. Patient participants were consecutively recruited from the outpatient clinic of the Aria Medical Complex for MS in Tehran, Iran. Patients were diagnosed by a consultant neurologist according to the McDonald diagnostic criteria (2010 revision) [40]. Healthy controls (HC) were recruited through local advertisements.

**Table 1:** Demographic and disease related information for participants in substudy 1

Characteristic	MS (***n = 91***)	HC (***n = 83***)	***p***-value
**Age –mean years** ± **SD**	37.24 ± 10.2	36 ± 10	0.42
**Education in years –mean** ± **SD**	14.21 ± 3.16	14.81 ± 2.5	0.16
**Gender (%female)**	75 (82%)	58 (70%)	0.052
**Disease Duration (in years)**	6.8		
**Disease course**			
**Relapsing remitting**	83 (91%)		
**Secondary progressive**	6 (7%)		
**Primary progressive**	2 (2%)		
**EDSS –mean** ± **SD**	1.27 ± 1.8		

*P*-values come from a two-sample t-test. MS: Multiple Sclerosis. HC: Healthy Controls. SD: standard deviation

**Table 2:** Demographic and disease related information for participants in Substudy 2

Number of Participants	Disease Course	Age- mean years ± SD	Gender (%female)	EDSS- mean ± SD	Education	Disease Duration
48 MS patients	Relapsing remitting	34.12 ± 8.5	31 (64%)	2.95 ± 0.92	14.79 ± 1.41	8.27 ± 5.20

Exclusion criteria: severe depression and other major psychiatric comorbidities, presence of neurological disorders and medical illness that independently affect brain function and cognition (other than MS for the patient group), visual problems that cannot be corrected with eye-glasses such that the problem prevents participants from reading, upper limb motor dysfunction, history of epileptic seizures, history of illicit substance and/or alcohol dependence.

For each participant, the clinical characteristics of MS subtype, information on age, education and gender were also collected. We quantified participant disability and disability progression over time by utilising the Expanded Disability Status Scale (EDSS).

For the purposes of this study, patients with severe abnormality in at least one of the BICAMS sub-tests (defined as 2 standard deviations (SD) below the norm) or with mild abnormality (defined as 1 SD below the norm) in at least two sub-tests of BICAMS were identified as cognitively impaired.

### Study procedures

#### Substudy 1

One hundred seventy-four participants (Table 1) took the iPad-based ICA test and the pen-and-paper BICAMS test, administered in random order. The same researchers who administered the BICAMS directed participants on how to take the ICA test. In this substudy, we investigated convergent validity of the ICA test with BICAMS, ICA’s test-retest reliability and the sensitivity and specificity of the ICA platform in detecting cognitive impairment in MS.

To measure test-retest reliability for the ICA test, a subset of 21 MS and 22 HC participants were called back after 5 weeks (± 15 days) to take the ICA test as well as the SDMT. The subset’s characteristics were similar to the primary set in terms of age, education and gender ratio. For both SDMT and the ICA, the same forms of the tests were used in the re-test session. Note that in the ICA test, while the images were the same, they were presented in a different random order in each administration.

#### Substudy 2

In this substudy, we investigated ICA’s correlation with the level of serum NfL in 48 MS patients (Table 2). Participants took both the ICA and the SDMT test, administered in random order. The ICA and SDMT were administered in the same session, but blood samples were collected in another visit with a gap of 2–3 days in between.

Blood samples were collected in tubes for serum isolation, then centrifuged at 3000 rpm for 20 min of blood draw, and finally placed on ice. Serum samples were measured at 1:4 dilution. NfL concentrations in serum were measured using a commercial ELISA (NF-light® ELISA, Uman Diagnostics, Umeå, Sweden). We used Anti NF-L monoclonal antibody (mAB) as a capture antibody and a biotin-labeled Anti NF-L mAB as the detection antibody. All samples were measured blinded. ELISA readings were converted to units per milliliter by using a standard curve constructed by calibrators (Bovine lyophilized NfL obtained from UmanDiagnostics).

Participants in Substudy 2 also attended an 8-week physical and cognitive rehabilitation program, details of which are reported in separate studies [41, 42]. The physical rehabilitation program included a combination of endurance and resistance exercises, with gradually increasing intensities over the 8-week period. The cognitive rehabilitation program included playing newly-developed games in a virtual reality (VR) environment, targeting sensorimotor integration, memory-based navigation and visual search. For the purpose of this study we measure pre- and post-rehabilitation ICA results for these group of participants, and the ICA correlation with NfL pre- and post-rehabilitation.

Participants were divided into a rehabilitation group of 38 individuals and a control group of ten; the control group only took the tests before and after these 8 weeks without attending the rehabilitation program. The rehabilitation group attended three sessions each week, each of them lasting about 70 min.

### Accuracy, speed and ICA summary score calculations

In the ICA, participants’ responses to each image and their reaction times (i.e. time between image onset and response) are recorded and used to calculate their overall accuracy and speed. Speed and accuracy are then used to calculate an overall summary score, called the ICA score.

***Accuracy*** is simply defined as the number of correct categorizations divided by the total number of images, multiplied by 100.

1 $$ Accuracy=\frac{number\ of\ correct\ categorizations}{total\ number\ of\ images}\times 100 $$

***Speed*** is defined based on participants’ response reaction times to images they categorized correctly.

2 $$ Speed=\left[100,100\times {e}^{\frac{- mean\ correct\  RT}{1025}+0.341}\right] $$

*RT: reaction time.*

*e: Euler’s number ~ 2.7182 … …*

Speed is inversely related to reaction time; the higher the speed, the lower the reaction time.

#### Preprocessing

We used a boxplot to remove outlier reaction times, before computing the ICA score. A boxplot is a non-parametric method for describing groups of numerical data through their quartiles; and allows for detection of outliers in the data. Following the boxplot approach, reaction times greater than q3 + w * (q3 - q1) or less than q1 - w * (q3 - q1) are considered outliers (where q1 is the lower quartile, and q3 is the upper quartile of the reaction times; and “w” is a ‘whisker’; w = 1.5). The number of reaction-time data points removed by the boxplot can vary case by case; if this number exceeds 40% of the observed images, the results are deemed invalid and a warning is shown to the clinician to repeat the test. In this study none of the participants faced such a warning. The maximum percentage of outliers was 15%, which happened in one of the MS patients.

The ***ICA score*** is a combination of accuracy and speed, defined as follows:

3 $$ ICA\  Score=\left(\frac{Speed}{100}\times \frac{Accuracy}{100}\right)\times 100 $$

### ICA’s artificial intelligence (AI) engine

**Fig. 2:**
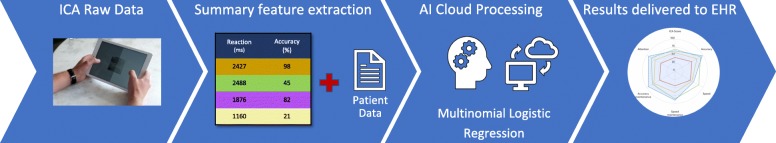
ICA’s AI’s pathway. The ICA measures categorization accuracy, processing speed, accuracy and speed over time and the raw data from these measurements are combined with patients’ demographic data, in order to provide a predictive score about participant’s cognitive status. The above-mentioned extracted features from the ICA raw data, plus patient’s demographic data are fed into an MLR classifier hosted on amazon AWS cloud services. The classifier then returns its predicted cognitive status, along with a probability, associated with the label, that shows how confident the AI engine is about the predicted label. The icons used in this figure are taken from Microsoft Office free icon library

ICA’s AI engine (Fig. 2) used in this study was a multinomial logistic regression (MLR) classifier trained based on a set of features extracted from the ICA test output for each participant. These features included the ICA score, and the trends of speed and accuracy during the test (i.e. whether the speed and/or accuracy were increasing or decreasing during the time-course of the test). The classifier also took subject’s age, gender and education in order to match subjects with similar demographics.

**Multinomial logistic regression classifier (MLR)** [43] is a supervised regression-based learning algorithm. The learning algorithm’s task is to learn a set of weights for a regression model that maps participants’ ICA test output to classification labels.

The difference between ICA’s AI engine in detecting cognitive impairment and the conventional way of defining a cut-off value for the outcome score of the test is further discussed in the discussion section.

## Results

### Convergent validity with BICAMS, and sensitivity to MS

**Fig. 3:**
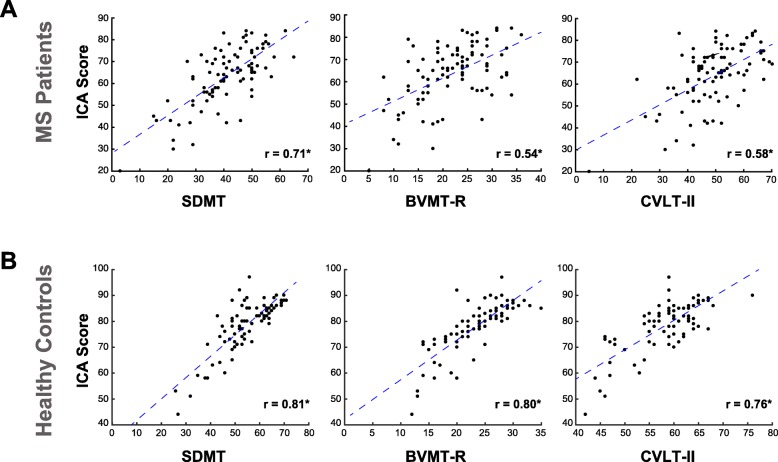
Correlation between BICAMS and ICA for (**a**) MS patients and (**b**) healthy controls. Each scatter plot shows the ICA score (y axis) vs. one of the tests in BICAMS (x axis). Each blue dot indicates an individual; the blue dashed lines are results of linear regression, fitting a linear line to the data in each plot. For each plot, the Pearson correlation between ICA and a BICAMS test is written in the bottom-right corner. If we combine the data from MS patients and healthy controls (*n* = 174 total), the ICA vs. BICAMS correlations will be the following: correlation with SDMT: 0.82 (*p* < 10–11); BVMT-R: 0.60 (*p* < 10–8); CVLT-II: 0.71 (*p* < 10–10). ICA: Integrated Cognitive Assessment; SDMT: Symbol Digit Modalities Test; BVMTR: Brief Visual Memory Test–Revised; CVLT-II: California Verbal Learning Test -2nd edition. Stars (*) show significant correlation at *p* < 10–8

In Substudy 1, we assessed convergent validity by examining the correlation between scores on the ICA test and the BICAMS battery (i.e. SDMT, BVMT-R and CVLT-II). Figure 3 presents scatterplots examining the relationship between BICAMS and ICA test performance. A high level of convergent validity is demonstrated between ICA and BICAMS. Within the BICAMS battery, SDMT had the highest correlation with the ICA test for the HC (Pearson’s *r* = 0.81, *p* < 10^− 14^), MS (*r* = 0.71, *p* < 10^− 13^), and combined (*r* = 0.82, *p* < 10^− 11^) groups. Scatterplots show ICA vs. BICAMS correlation separately for MS and HC; combining results from both groups (*n* = 174 total), we find a correlation of 0.82 with SDMT (*p* < 10^− 15^), 0.71 with CVLT-II (*p* < 10^− 10^), and 0.60 with BVMT-R (*p* < 10^− 8^). The correlation results between BICAMS and ICA are largely similar when including only relapsing-remitting MS patients (RRMS) [r (SDMT) =0.71 (*p* < 10–13); r (BVMT-R) = 0.51 (*p* < 10–6); r (CVLT-II) = 0.56 (*p* < 10–7)]. Correlations between ICA’s speed and accuracy components with the BICAMS battery are also reported in Table 3. Furthermore, we calculated BICAMS composite score by averaging the z-scores of the CVLT-II, the BVMT-R, and the SDMT. ICA had a correlation of *r* = 0.82 (*p* < 10^− 11^) with BICAMS composite score.

**Table 3:** Speed and accuracy correlations with BICAMS

	SDMT	BVMT-R	CVLT-II
**Speed**	*r* = 0.66 *	*r* = 0.42 *	*r* = 0.52 *
**Accuracy**	*r* = 0.55 *	*r* = 0.46 *	*r* = 0.52 *

Pearson correlations between the BICAMS battery and speed, accuracy components of the ICA test across all participants. (* shows statistical significance at *p* < 10^−6^)

To compare the sensitivity of the BICAMS and ICA in detecting MS dysfunctions, we compared mean test scores in MS and HC groups separately for BICAMS battery of tests and the ICA test (Table 4). Within the BICAMS battery, SDMT and CVLT-II could differentiate between HC and MS patients (Table 4). The scores on both SDMT and CVLT-II were significantly lower for the MS patients compared to the HC group. However, there was no significant difference between BVMT-R scores of the HC and MS groups. These results are consistent with previous findings showing that SDMT has a better sensitivity in detecting MS compared to other tests within the BICAMS battery [20, 44]. We repeated these analyses for the subset of RRMS patients (Supplementary Table 1), the results of which were similar to when all patients were included.

**Table 4:** Mean ICA and BICAMS scores per group

	MS ***(n = 91)***		HC ***(n = 83)***				
BICAMS	mean	SD	mean	SD	Difference	Cohen’s d	***p***-value
SDMT	41.04	11.02	54.73	9.77	13.69	1.31	< 10^− 14^
BVMT-R	21.89	6.95	23.69	5.17	1.80	0.29	=0.0565
CVLT-II	48.96	11.13	58.28	6.59	9.32	1.00	< 10^−9^
**ICA**							
ICA score	63.67	13.30	78.43	9.86	17.76	1.26	< 10^−13^
Accuracy	84.97	11.66	89.57	5.79	4.60	0.50	=0.0014
Speed	74.54	12.26	87.76	10.11	13.22	1.16	< 10^−12^

Mean and standard deviations (SD) for BICAMS test scores and the ICA test scores are compared for MS patients versus healthy controls (HC). The ICA score is a composite score made of both speed and accuracy of participants in ICA’s rapid visual categorization task. *P*-values come from a two-sample t-test

As shown in Table 4, the ICA could discriminate between HC group and MS patients, at least as accurately as the SDMT; however, the ICA, as a digital test, has the advantages described in Fig. 8.

Given that the ICA test involves tapping left or right on an iPad, we investigated the relation between handedness and ICA score. The correlation between ICA score and handedness was *r* = − 0.13, *p* = 0.07 (not significant), which is comparable (and lower) than the correlation between handedness and SDMT’s score (*r* = − 0.17).

### ICA accuracy in detecting cognitive impairment

**Fig. 4:**
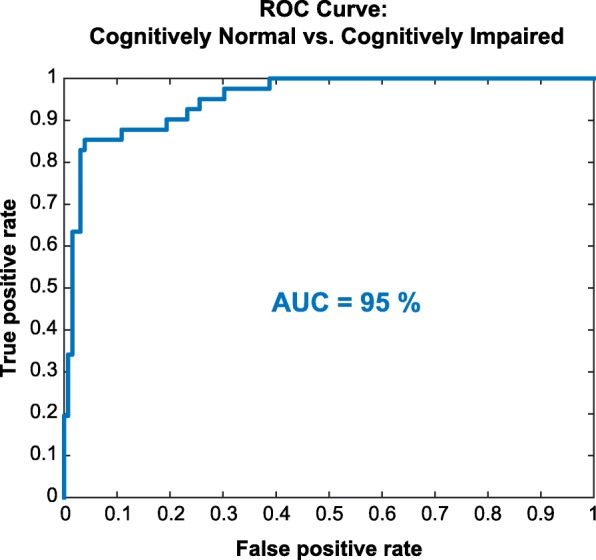
ROC curve for the ICA test in discriminating cognitively impaired from cognitively healthy individuals. A multinomial logistic regression classifier was trained based on the ICA test output, and tested using leave-one-out cross-validation. AUC =95.1%; Sensitivity = 82.9%; Specificity = 96.1%

45% of MS patients were identified to have cognitive impairment. Using an ROC curve (Fig. 4), we then assessed the accuracy of the ICA’s AI engine (i.e. MLR classifier) in discriminating cognitively healthy from cognitively impaired individuals (Fig. 4, area under curve (AUC) = 95.1%, sensitivity = 82.9%, and specificity = 96.1%.)

### ICA and SDMT correlations with Neurofilament light (NfL)

**Fig. 5:**
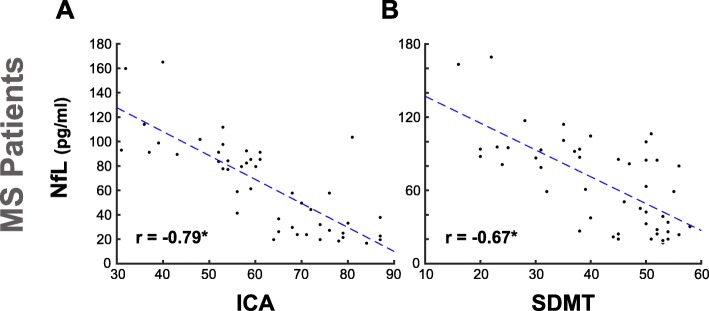
ICA correlation with severity of neural damage, as measured by serum NfL. Each scatter plot shows the NfL level in serum (y axis) vs. ICA or SDMT (x axis). Each blue dot indicates an individual; the blue dashed lines are results of linear regression, fitting a linear line to the data in each plot. For each plot, the Pearson correlation between NfL level and the reference cognitive test is written in the bottom-left. Stars (*) show significant correlations at *p* < 10^− 6^

NfL is a promising fluid biomarker of disease monitoring for various brain disorders, such as Alzheimer’s Disease and Multiple Sclerosis [45, 46] . In Substudy 2, we demonstrated that there is a strong correlation between ICA score and the level of serum NfL (*r* = − 0.79, *p* < 10^− 10^) (Fig. 5a). For comparison, on the same set of MS participants, SDMT correlations with NfL is also reported (*r* = − 0.67, *p* < 10^− 6^) (Fig. 5b). SDMT and ICA were both administered in the same session.

### ICA’s performance pre- and post- rehabilitation

**Fig. 6:**
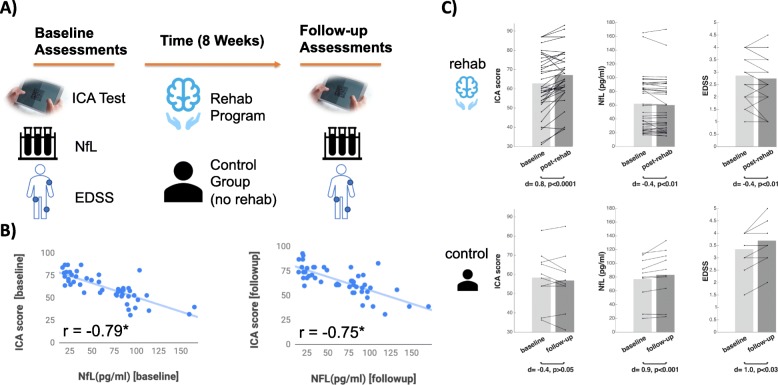
**a** Participants were divided into the rehabilitation group and the control group. All participants were assessed with ICA, serum NfL and their EDSS score at the baseline and after 8 weeks. **b** ICA had a significant correlation of r = − 0.79 (*p* < 10^− 10^) with NfL at baseline (also reported in Fig. 5a), and a significant correlation of *r* = − 0.75 (*p* < 10^− 8^) after the 8 weeks. **c** The bars indicate the average ICA, EDSS and the level of NfL at the baseline, and after the 8 weeks separately for each group of participants. Connected lines from the light gray bars (baseline) to dark gray bars (follow-up) show the changes in score for each individual. The difference between the two bars are reported in Cohens’ d below each pair of the bar graphs. The icons used in this figure are taken from Microsoft Office free icon library

We examined the level of serum NfL pre- and post- rehabilitation, as well as patients ´ EDSS and ICA scores (Fig. 6). In the rehabilitation group, after the 8-week rehabilitation program, we observed a significant increase in ICA score (Cohen’s d = 0.8, *p* < 0.0001), and a significant decrease in serum-NfL (d = − 0.4, *p* < 0.01) and EDSS score (d = − 0.4, *p* < 0.01). In the control group, we found the opposite pattern after 8 weeks, that is a decrease in ICA score (d = − 0.4, *p* > 0.05) and a significant increase in serum-NfL (d = 0.9,*p* < 0.001) and EDSS score (d = 1.0, *p* < 0.03).

### ICA test-retest reliability and absence of a learning bias

Test-retest reliability was measured by computing the Pearson correlation between the two ICA scores. R values for test-retest correlation are considered adequate if > 0.70 and good if > 0.80 [47].

**Fig. 7:**
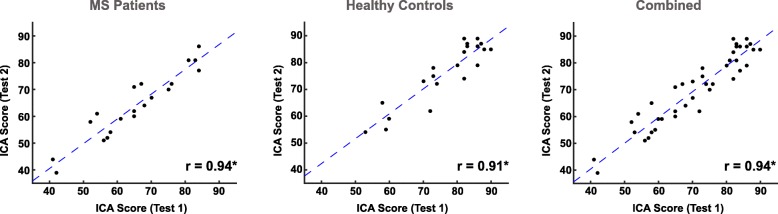
Test-retest reliability scatter plots for the ICA test. Scatterplots are presented comparing ICA scores at Time 1 versus Time 2 administrations for the MS, HC, and combined groups. The gap between the 1st and the 2nd administration of the ICA test was 5 weeks (+ − 15 days). Reliability is calculated using Pearson’s r. The test-retest reliability for the SDMT test was: r (combined) = 0.97; r (HC) = 0.98; r (MS) = 0.97. Stars (*) indicate statistical significance at *p* < 10^− 8^

Figure 7 presents scatterplots of ICA performance comparing 1st administration versus 2nd administration of the test for the HC, MS, and combined groups. Test–retest reliability was high, with correlation values in the range between 0.91 and 0.94.

In the subgroup of participants (21 MS and 22 HC) who took the ICA and SDMT for a second time, we studied whether they could perform better because of a previous exposure to either of the tests. This is called a learning bias (also referred to as a practice effect). As shown in Table 5, comparing the first and second administration of the ICA and SDMT tests, ICA showed no learning bias. However, we see an improvement in participants’ average SDMT score. This improvement in SDMT score (i.e. learning bias) was statistically significant in the HC group, but not in the MS group.

**Table 5:** Learning bias (practice effect) for ICA and SDMT

Group	Test Name	Test 1 [mean ± SD]	Test 2 [mean ± SD]	Paired t-test		
				difference	Cohen’s d	***p***-value
**MS (*n = 21*)**	**ICA**	64.76 ± 12.7	64.66 ± 12.2	− 0.09	− 0.02	0.91
	**SDMT**	43.6 ± 10	44.2 ± 11	0.66	0.25	0.25
**HC (*n = 22*)**	**ICA**	77.04 ± 11	76.86 ± 11	−0.18	− 0.07	0.73
	**SDMT**	55.13 ± 12	56.36 ± 12.3	1.22	0.50	0.02*

Mean and standard deviations (SD) of SDMT and ICA scores are compared between the two administrations of the tests for each group of participants. Only SDMT in healthy subjects showed a significant learning bias

### ICA correlation with EDSS, age and education

To further characterize the ICA score and its relationship with other measures from the MS patients, we calculated the correlation between ICA score and patients’ EDSS, age and education (Table 6). Both BICAMS and ICA scores were negatively correlated with patients’ EDSS, demonstrating an inverse relation between disability scale and cognitive performance. For all the tests, we also observed a decrease in performance as age increased, showing the effect of aging on cognitive performance. All tests were correlated with participants’ level of education, with ICA having the lowest correlation.

**Table 6:** Age/EDSS/Education vs. BICAMS/ICA

		BICAMS			
		SDMT	BVMT-R	CVLT-II	ICA
**Correlation with**	**EDSS**	−0.41 (*p* < 10^−4^)	− 0.26 (*p* < 0.05)	−0.33 (*p* < 0.001)	− 0.58 (*p* < 10^−8^)
	**Education**	0.50 (*p* < 10^−6^)	0.34 (*p* < 0.001)	0.31 (*p* < 0.01)	0.25 (*p* < 0.05)
	**Age**	−0.38 (*p* < 10^−4^)	− 0.50 (*p* < 10^−5^)	−0.42 (*p* < 10^− 4^)	−0.49 (*p* < 10^− 6^)

The table shows Pearson Correlations of the ICA score and the BICAMS battery of tests with MS patients’ EDSS score, education in years, and their age. *EDSS* Expanded Disability Status Scale; *BICAMS* Brief International Cognitive Assessment for MS; *SDMT* Symbol Digit Modalities Test; *BVMT-R* Brief Visual Memory Test–Revised; *CVLT-II* California Verbal Learning Test -2nd edition; *ICA* Integrated Cognitive Assessment

## Discussion

In this validation study, we demonstrate that the ICA test has convergent validity with BICAMS, with an excellent test-retest reliability comparable to that reported for SDMT [10]. In the ICA test, comparing speed versus accuracy (Table 4), speed seems to play a more significant role in discriminating MS patients from HC participants. This corroborates findings from other studies suggesting slower speed of information processing as a key deficit in multiple sclerosis [48]. IPS impairment also underlies other areas of cognitive dysfunction [15, 49]. This is because the speed with which an individual performs a cognitive task is not simply an isolated function of the processes required in that task, but also a reflection of their ability to rapidly carry out many different types of processing operations. In the case of ICA, these operations include transferring visual information through retina to higher level visual areas (i.e. sensory speed), processing the image representation in the visual system to categorize it into animal or non-animal (i.e. cognitive speed), and then translating this into a motor response (i.e. motor speed).

We also explored the link between disability (as measured by EDSS) and cognitive impairment. Patients with cognitive impairment are typically found to be at higher risk of developing further disability [6, 8]. While we did not carry out a long-term monitoring of disability progression in our patients in this study, the negative correlation between ICA score and EDSS score corroborates previous findings that lower cognitive performance is linked with higher disability (i.e. as indicated by higher EDSS scores).

In contrast to most of the currently standard cognitive tests, whereby stimuli are language-dependent, the presented stimuli in the ICA test are natural images that contain universally recognizable images of animals or objects, thus making the test intrinsically language-independent. Furthermore, participants’ responses only involve tapping on the left or right side of an iPad, making it totally independent of participants’ knowledge of Arabic numerals or alphabet and words, or ability of a participant to draw shapes (as in BVMT-R). This makes the ICA test more suitable for wider international use, and less dependent on linguistic, educational, and demographic differences.

**Fig. 8:**
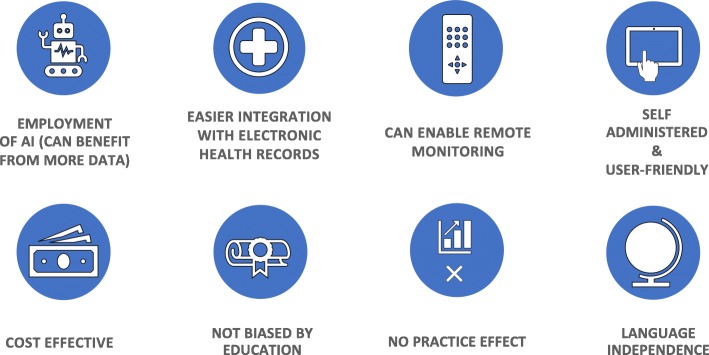
ICA key features as a computerized test. Eight key attributes of the ICA test that can save expensive clinical time and make the test scalable and more accessible to wider populations; as well as its capability to use new data as they become available to improve its reliability over time. The icons used in this figure are taken from Microsoft Office free icon library

Computerized tests have several advantages over pen-and-paper tests, such as a) efficient administration that can save expensive clinical time, b) automatic scoring, which reduces errors in calculating and transferring scores, and c) easier integration with electronic medical records or research databases. The use of digital technology in this context can reduce barriers for both clinicians and patients to deliver or receive the assessments that would benefit their treatment and health throughout the course of the disease. With ICA, we aimed to develop a test that can close the current gap in clinical practice between patients’ needs and what clinicians can offer in terms of the much-needed routine cognitive assessment and disease monitoring. Such a test must have a certain set of attributes, in addition to being sensitive and accurate. Figure 8 summarizes some of the key attributes of the ICA test, as a computerized test, that has made it more scalable, accessible and cost-effective compared to the standard pen-and-paper tests. In addition, the ICA has some unique features that are absent from the computerized versions of the standard cognitive tests, such as the ability to benefit from more data to improve its performance over time. One basic difference between the ICA’s classification of patients (using the AI engine) and the conventional way of defining an optimal cut-off value for classification is the dimensionality (or the number of features) used to make the classification. For example, in a conventional assessment tool, an optimal cut-off value is defined based on the test score. This is a one-dimensional classification problem, and there is only one free parameter to optimize, resulting in less flexibility to learn from more data. In contrast, the ICA generates a richer pool of data (one reaction time and accuracy result per image). Although the ICA score is the most informative summary score, the use of a classifier enabled us to find the optimum classification boundary in the higher dimensional space. With more free parameters to optimize, the classifier benefited from more data to set these parameters to achieve a higher classification accuracy. The ICA’s performance can be further improved over time through new batches of training on additional data to update its cloud-based AI model.

It is worth noting that while electronic implementations of the SDMT [50, 51] do exist, they retain two significant differences from the ICA. First, the ICA takes advantage of an AI platform, and thus the capacity to benefit from big data, as explained before. Second, the ICA did not show a learning bias in this and a previous study [19], as opposed to the learning bias reported for the iPad-based SDMT (i.e. PST) [50].

To make an early diagnosis of MS and monitor disease activity, reliable biomarkers are required. In Substudy 2, we demonstrated a strong association between ICA score and NfL in MS patients. This is particularly of interest given the totally non-invasive nature of the ICA test, and its easy and inexpensive scalability for administration in large populations. The 8-week follow-up of the rehabilitation group, compared to the control group, further shows ICA’s sensitivity to track changes in cognition. For frequent cognitive assessments, digital biomarkers have an advantage over fluid biomarkers, given their lower cost, accessibility, the possibility of remote administration and easier integration into routine clinical practice.

Limitations encountered in this study include the lack of NfL data from the healthy control group, and the absence of neuroimaging markers of disease activity. We acknowledge the relatively short follow-up period of patients post-rehabilitation. Future studies are needed to investigate the link between ICA test results and other measures of brain atrophy, in particular, given the strong link between ICA and NfL—which reflects neural damage—would be informative to investigate the ICA relation with cortical thickness in MS patients.

## Conclusions

Overall, our results provide evidence for the use of the ICA in clinical practice as an accurate tool for assessing cognitive impairment in MS. Digital biomarkers of cognition (such as ICA) can be used to monitor the progress of cognitive impairment, which subsequently paves the way for using cognition as a marker of disease activity in MS. The ICA has the potential to be used as a high-frequency monitoring tool of treatment efficacy both in the clinic and remotely such as at patients’ homes. Future longitudinal studies need to test these hypotheses in larger patient samples.

## Supplementary information

**Additional file 1.** Supplementary Table 1 Mean ICA and BICAMS scores for RRMS and HC

## Data Availability

De-identified raw data are available to qualified investigators upon reasonable request from the corresponding author for the purposes of replicating procedures and results.

## Abbreviations

MS: Multiple sclerosis
AI: Artificial intelligence
ICA: Integrated cognitive assessment
BICAMS: Brief international cognitive assessment for multiple sclerosis
HC: Healthy controls
NfL: Neurofilament light
IPS: Information processing speed
SDMT: Symbol digit modalities test
PST: Processing speed test
CVLT-II: California verbal learning test -2nd edition
BVMT-R: Brief visual memory test–revised
ISI: Inter-stimulus interval
