# Supplementary material for: A self-administered, artificial intelligence (AI) platform for cognitive assessment in multiple sclerosis (MS)
Source: BMC Neurol. 2020 May 18;20:193. doi: 10.1186/s12883-020-01736-x (PMC7236354; doi:10.1186/s12883-020-01736-x)
Supplement: Supplementary file 1 — Additional file 1. Supplementary Table 1 Mean ICA and BICAMS scores for RRMS and HC [file 12883_2020_1736_MOESM1_ESM.docx]

**Supplementary Table 1. Mean ICA and BICAMS scores for RRMS and HC**

|  | RRMS *(n=83)* | | HC *(n=83)* | | |  |  |  |
| --- | --- | --- | --- | --- | --- | --- | --- | --- |
| BICAMS | mean | SD | mean | | SD | Difference | Cohen’s d | p-value |
| SDMT | 41.77 | 10.78 | 54.73 | | 9.77 | 12.96 | 1.26 | <10^-13^ |
| BVMT-R | 22.38 | 6.87 | 23.69 | | 5.17 | 1.31 | 0.22 | =0.17 |
| CVLT-II | 49.75 | 11.01 | 58.28 | | 6.59 | 8.53 | 0.94 | <10^-8^ |
| ICA |  | | |  | | | | |
| ICA score | 65.24 | 12.31 | 78.43 | | 9.86 | 13.19 | 1.18 | <10^-13^ |
| Accuracy | 86.17 | 10.38 | 89.57 | | 5.79 | 3.4 | 0.40 | <0.01 |
| Speed | 75.47 | 10.73 | 87.76 | | 10.11 | 12.29 | 1.18 | <10^-11^ |
